# Thymic Stromal Lymphopoietin Promotes MRGPRX2-Triggered Degranulation of Skin Mast Cells in a STAT5-Dependent Manner with Further Support from JNK

**DOI:** 10.3390/cells10010102

**Published:** 2021-01-08

**Authors:** Magda Babina, Zhao Wang, Kristin Franke, Torsten Zuberbier

**Affiliations:** Mast Cell Biology Unit, Department of Dermatology and Allergy, Charité–Universitätsmedizin Berlin, Corporate Member of Freie Universität Berlin, Humboldt-Universität zu Berlin, and Berlin Institute of Health, 10117 Berlin, Germany; zhao.wang@charite.de (Z.W.); kristin.franke@charite.de (K.F.); torsten.zuberbier@charite.de (T.Z.)

**Keywords:** mast cell, MRGPRX2, pseudo-allergy, Substance P, TSLP, skin

## Abstract

Thymic stromal lymphopoietin (TSLP) is released by epithelial cells following disturbed homeostasis to act as “alarmin” and driver of Th2-immunity. Aberrant TSLP expression is a hallmark of atopic diseases, including atopic dermatitis (AD). Mast cells (MCs) are overabundant in AD lesions and show signs of degranulation, but it remains unknown whether TSLP contributes to granule discharge. Degranulation of skin MCs proceeds via two major routes, i.e., FcεRI-dependent (allergic) and MRGPRX2-mediated (pseudo-allergic/neurogenic). Evidence is accumulating that MRGPRX2 may be crucial in the context of skin diseases, including eczema. The current study reveals TSLP as a novel priming factor of human skin MCs. Interestingly, TSLP selectively cooperates with MRGPRX2 to support granule discharge, while it does not impact spontaneous or FcεRI-driven exocytosis. TSLP-assisted histamine liberation triggered by compound 48/80 or Substance P, two canonical MRGPRX2 agonists, was accompanied by an increase in CD107a+ cells (a MC activation marker). The latter process was less potent, however, and detectable only at the later of two time points, suggesting TSLP may prolong opening of the granules. Mechanistically, TSLP elicited phosphorylation of STAT5 and JNK in skin MCs and the reinforced degranulation critically depended on STAT5 activity, while JNK had a contributory role. Results from pharmacological inhibition were confirmed by RNA-interference, whereby silencing of STAT5 completely abolished the priming effect of TSLP on MRGPRX2-mediated degranulation. Collectively, TSLP is the first factor to favor MRGPRX2- over FcεRI-triggered MC activation. The relevance of TSLP, MCs and MRGPRX2 to pruritis and atopic skin pathology indicates broad repercussions of the identified connection.

## 1. Introduction

Acute mast cell (MC) activation leads to hypersensitivity reactions and is the root of disorders like rhinoconjunctivitis, asthma, urticaria, angioedema, food allergy, and anaphylaxis [1,2]. In these conditions, degranulation leads to multiple symptoms in the respective organs caused by the mediators rapidly secreted from MCs, especially histamine. Clinically relevant MC activation can be evoked by the allergic route involving FcεRI, IgE, and allergen, or by the more recently discovered MAS-related G protein coupled receptor-X2 (MRGPRX2)-dependent pathway. The latter receptor can be activated by a wide range of agonists, including neuropeptides (e.g., Substance P) and exogenous substances (like compound 48/80, antibiotics, opiates) [3,4,5,6,7,8]. The efficient triggering of MC degranulation and the large number of ligands have moved MRGPRX2 to the center of attention in recent years [9,10,11,12,13,14]. In fact, the receptor is now widely believed to constitute a major participant in drug-induced pseudo-allergic/anaphylactoid phenomena and in MC-dependent diseases triggered by exogenous or endogenous neuro-, host defense, and other peptides [4,6,15,16].

Only certain MC-subtypes, especially those in the skin (so-called MCTC-type MCs) express MRGPRX2 at high level [17,18,19,20], while MCs in most other organs do not express the receptor, and hence are refractory to stimulation by its ligands [21,22,23]. Skin MCs are thus the most adequate system to explore MRGPRX2 regulation and function, as they are also the most likely responders to MRGPRX2 ligands in physiological and pathological contexts in vivo.

The allergic and pseudo-allergic pathways differ fundamentally in several aspects, including spatiotemporal patterns of granule discharge and dependence on IKK-β, the latter involved in SNAP23/STX4 complex formation, which is key to the so-called compound exocytosis observed following FcεRI aggregation, in which granules fuse together and are released as bigger, more heterogeneously shaped entities [24]. Interestingly, several typically supportive factors of the MC lineage can curb MRGPRX2 function, as highlighted by SCF [18,19], the most universal growth factor of the lineage [11,25,26,27]. This likewise applies to a certain extent to IL-4 [19] and IL-33, the latter dampening MRGPRX2 expression and thereby curtailing its function [28]. Retinoic acid, regulating a number of functions in the MC lineage [29], likewise dampens the MRGPRX2-dependent route [30]. It is of note that all but IL-33 influence FcεRI-dependent stimulation in the opposite way, indicating that several conditions can favor one route over the other. An exception is IL-33 which, when provided as an acute stimulus, supports degranulation triggered via both FcεRI and MRGPRX2 [28].

Together with IL-33, thymic stromal lymphopoietin (TSLP) is widely recognized as a critical factor in allergic disorders, including rhinitis, asthma, food allergy, eosinophilic esophagitis, and atopic dermatitis (AD), i.e., conditions, in which MCs act as critical effector cells [31,32,33,34,35,36]. As a consequence, the anti-TSLP antibody tezepelumab is in clinical trials for asthma and AD [37,38].

Both IL-33 and TSLP belong to the subgroup of epithelial-derived innate cytokines that support Th2 responses in part by re-programming of dendritic cells and support of innate lymphoid cells [35,39,40,41]. Interestingly, however, MCs were the most efficient producers of the TSLPR transcript (gene: CRLF2) in the FANTOM5 (Functional annotation of the mammalian genome 5) atlas, a transcriptome collection of roughly 1800 cell and tissue samples from all across the body, pointing towards MCs as meaningful target cells [17,42,43]. Following up on this finding, we recently reported that TSLP efficiently maintains survival of skin MCs by acting through a complex mechanism composed of two parallel axes, i.e., STAT5/Mcl-1 and JNK/Bcl-xl [44]. While being renowned for its Th2 skewing potential, TSLP can, in addition to these more long-term effects, act as an epithelial alarmin, that is swiftly synthesized and released upon tissue damage, including microbial challenge, trauma, and physical or chemical insults [45,46,47]. Under these circumstances, TSLP can act acutely, and MCs are ideally positioned in tissues to swiftly respond to and integrate signals from infectious agents, allergens, drugs and tissue-derived alarmins.

TSLP, cutaneous MCs, FcεRI and, more recently, MRGPRX2 have all been linked to AD pathogenesis [9,36,48], and we theorized that these elements may be inter-connected. We now report that TSLP can indeed modulate the secretory competence of skin MCs. Surprisingly, however, only MRGPRX2-evoked degranulation was promoted by TSLP, whereas allergic stimulation remained unaffected. In addition to histamine release, TSLP promoted CD107a exteriorization, yet to a lesser extent. Mechanistically, TSLP support was orchestrated by the dominant action of STAT5 with further support from JNK, the two cascades most prominently activated by TSLP in skin MCs [44].

## 2. Materials and Methods

### 2.1. Skin Samples

This was an unlinked anonymous study. Donor skins, which otherwise would be disposed of, were obtained from circumcisions (foreskin), with written informed consent of the patients or their legal guardians, as in previous studies from our group [18,19,28,44]. The study was approved by the Ethics Committee of the Charité Universitätsmedizin Berlin and experiments were conducted according to the Declaration of Helsinki Principles.

### 2.2. Skin MC Purification and Culture

MCs were isolated from skin samples by a routine procedure, as described [49,50]. Briefly, foreskin samples from 2 to 10 donors (most commonly 3–7) were pooled, the skin cut into strips and treated with dispase (BD Biosciences, Heildelberg, Germany) at 3.5 U/mL and 4 °C overnight. The epidermis was removed, the dermis was chopped and digested with 1.5 mg/mL collagenase type 1 (Worthington, Lakewood, NJ, USA), 0.75 mg/mL hyaluronidase type 1-S (Sigma, Steinheim, Germany), and DNase I at 10 μg/mL (Roche, Basel, Switzerland) at 37 °C in a shaking water bath for 75 min. The cells were separated from remaining tissue by filtration. MC purification was achieved by positive selection with anti-human c-Kit microbeads and the Auto-MACS (both from Miltenyi Biotec, Bergisch Gladbach, Germany). MC purity always exceeded 98%, as assessed by acidic toluidine-blue staining. MC culture was performed as described previously [17,51,52]. In brief, cells were cultured at 5 × 10^5^/mL in Basal Iscove medium with 10% FCS (Biochrom, Berlin, Germany) for around 3 weeks. SCF (Peprotech, Rocky Hill, NJ, USA) (at 100 ng/mL) and IL-4 (Peprotech) (10 ng/mL) were provided twice a week.

### 2.3. Histamine Release Assay (HRA)

The HRA was performed according to a method routinely employed in our laboratory [5,53]. In brief, MCs in PAG-CM buffer (Piperazine-N,N-bis [2-ethanesulfonic acid]-Albumin-Glucose buffer containing 3 mM CaCl2 and 1.5 mM MgCl2, pH 7.4) were stimulated by FcεRI-aggregation (anti-AER-37 antibody at 0.2 µg/mL eBioscience, San Diego, CA, USA), or c48/80 (Sigma, at 10 µg/mL), or Substance P (SP) (Bachem, Budendorf, Switzerland at 30 µM), or no stimulus (spontaneous) for 30 min at 37 °C. Priming with TSLP (7.5 ng/mL) versus PAG-CM (buffer control) was performed for 30 min before stimulation. Histamine in the supernatants was measured by an automated fluorescence method (Alliance Instruments, Salzburg, Austria). Total cellular histamine content was measured analogously. All determinations were performed in triplicate. Net histamine release (%) was calculated as ((stimulated release–spontaneous release)/complete histamine in the MC preparation) × 100.

For inhibitor studies in the absence of TSLP, cells were pretreated with SP600125 at 5 µM (JNK inhibitor) or Pimozide at 5 µM (STAT5 inhibitor) versus PAG-CM buffer (control) for 15 min and then stimulated with the above stimuli for 30 min.

To study the involvement of selected kinases in TSLP’s potentiation of degranulation, cells were pretreated with the above inhibitors for 15 min, TSLP was added to selected vials, and finally the secretagogues (AER-37 or MRGPRX2 ligands) were added as above.

### 2.4. Accell^®^ Mediated RNA Interference (RNAi)

RNA interference in MCs was performed according to an established protocol by using the Accell^®^ siRNA technology (Dharmacon, Lafayette, CO, USA) [28,44,54]. Briefly, MCs were washed with Accell siRNA medium (supplemented with Non-Essential Amino Acids and L-Glutamine), plated at 1 × 10^6^/mL in Accell siRNA medium and treated with 1 µM STAT5-targeting siRNA (E-005169-00-0050), or JNK-targeting siRNA (E-003514-00-0050) or non-targeting siRNA as control (D-001910-10-50) for 48 h. After incubation, cells were primed with or without TSLP and degranulation was induced by MRGPRX2 ligands as above.

### 2.5. β-Hexosaminidase Release Assay

β-hexosaminidase assays were run exactly as described [28,50]. Briefly, cell suspensions were washed, resuspended at 5 × 10^5^ cells/mL in PAG-CM buffer. Aliquots of 100 μL were stimulated by compound 48/80 (10 μg/mL), or SP (30 µM), or kept in buffer only. After incubation for 60 min, supernatants (SNs) were collected by centrifugation at 500× *g*, 4 °C for 3 min, and the pelleted MCs rapidly frozen at −80 °C. After thawing, aliquots of 50 μL of 4-methyl umbelliferyl-N-acetyl-beta-D-glucosaminide (Sigma-Aldrich, Munich, Germany) solution at 5 μM in citrate buffer (pH 4.5) were mixed with the same volume of supernatant or lysate and incubated for 60 min at 37 °C. The reaction was stopped by adding 100 mM sodium carbonate buffer (pH 10.7). Fluorescence intensity was measured at an emission wavelength of 460 nm after excitation at 355 nm. Percent β-hexosaminidase release was calculated as (fluorescence intensity SN/(fluorescence intensity SN + fluorescence intensity lysate)) × 100. Net release was calculated by subtracting spontaneous release, as in the histamine release assay above.

### 2.6. Flow Cytometry

Flow-cytometric detection of CD107a cell surface expression was as described [5,55]. In brief, MCs were pre-stimulated with or without TSLP (7.5 ng/mL) for 30 min, then subjected to 0.2 µg/mL FcεRI-aggregation (anti-AER-37 antibody for 15 min, 30 min, 60 min), 10 µg/mL c48/80 or 30 µM SP (for 2 min and 8 min), or no stimulus (control). The reaction was stopped by ice-cold 4% paraformaldehyde for 15 min. After washing, the cells were incubated with 10 µL of anti-human CD107a-FITC antibody (LAMP-1) (BioLegend, San Diego, CA, USA) together with 10 µL of human AB-serum for 30 min at 4 °C, then washed and CD107a expression was detected by the Facscalibur (BD Biosciences, San Jose, CA, USA).

MRGPRX2 cell surface staining was performed according to established protocols [5,28]. In brief, cells were blocked for 15 min with human AB-serum, incubated with anti-human MRGPRX2 (clone K125H4, Biolegend, 0.15 µg/mL) or isotype mouse IgG2b-PE (clone eBMG2b, eBioscience) for 30 min at 4 °C, and analyzed as above.

Intracellular staining of signaling intermediates was performed exactly as described [44]. In brief, MCs were incubated for 30 min with TSLP, then fixed with 4% paraformaldehyde and permeabilized with Saponin prior to being stained with anti-pSTAT5 or anti-pJNK primary antibodies or the respective isotype control (both from Cell Signaling Technologies, Danvers, MA, USA), followed by incubation with a PE-labeled secondary antibody (Jackson Immunoresearch, Cambridgeshire, UK).

All data were analyzed with the FlowJo 7.6.5 analysis software (FlowJo LLC, Ashland, OR, USA).

### 2.7. Immunoblotting

Cell preparation and immunoblotting were performed as described [44]. In brief, MCs were incubated or not with TSLP for 30 min, lysed and lysates separated through 4–12% Bis-Tris Gel (Thermofisher, Bleiswijk, The Netherlands) SDS-PAGE, the proteins transferred to nitrocellulose membranes, the membranes blocked with 1× casein blocking buffer (Sigma Aldrich, St. Louis, MO, USA) and incubated with primary antibodies against phospho- and total-STAT5 as well as ß-actin (all from Cell Signaling Technologies) overnight and subsequently with horseradish peroxidase-conjugated secondary antibodies (Merck Millipore, Darmstadt, Germany). Blots were developed, and bands visualized by a chemiluminescence assay (Weststar Ultra 2.0, Cyanagen, Bologna, Italy) according to the manufacturer’s instructions, and the bands were recorded using a detector for chemiluminescence (Fusion FX7 Spectra, Vilber Lourmat, Eberhardzell, Germany). Densitometric measurements were assessed by the software ImageJ (National Institutes of Health, Bethesda, MD, USA) and arbitrary values were determined as relative target expression (densitytarget protein/densityloading control protein); relative target expression of control cells was set as 1.

### 2.8. Statistical Analysis

Statistical analyses were performed using PRISM 8 (GraphPad Software, La Jolla, CA, USA). Pairwise comparisons of TSLP priming versus control were conducted by Student’s t-test. One sample t-test (against 1) was used to calculate significance in normalized immunoblot data. For multi-group comparisons, RM one-way ANOVA with Sidak’s multiple comparisons test were applied. Fold changes (FC) of histamine and CD107a were calculated as net “parameter” after TSLP priming/net “parameter” without TSLP priming, whereby parameter refers to percent histamine release or the proportion of CD107a+ cells. The resulting FC (fold change) values were compared by Mann–Whitney test. *p* < 0.05 was considered statistically significant.

## 3. Results

### 3.1. TSLP Primes Human Skin-Derived MCs for Enhanced MRGPRX2-Elicited Degranulation

So far, a positive impact of TSLP on MC cytokine production was reported [56,57,58] and TSLP was also found to stimulate PGD2 from cord-blood-derived MCs [59].

The most selective MC function, i.e., degranulation with the release of prefabricated histamine and other mediators confined to these cells, has not been investigated or not found to be modulated by TSLP [60].

**Figure 1:**
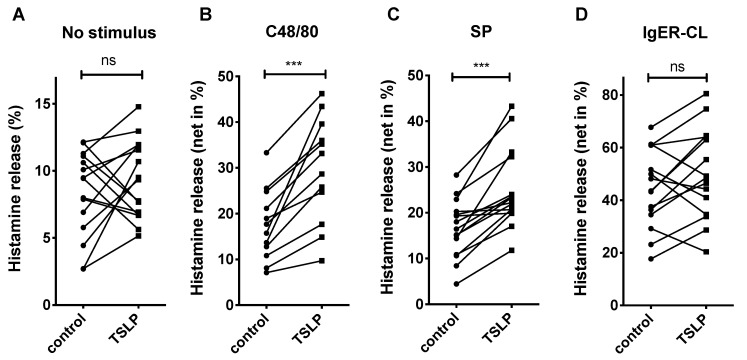
Thymic stromal lymphopoietin (TSLP) priming selectively promotes MRGPRX2-mediated histamine release in human skin mast cells (MCs). Skin-derived MCs were pre-stimulated with TSLP (7.5 ng/mL) or vehicle (control) for 30 min prior to stimulation. Histamine release was assessed 30 min after treatment with (**A**) no stimulus; (**B**) c48/80 (10 µg/mL); (**C**) Substance P (SP) (30 µM); (**D**) IgER-CL (IgER-crosslinking; AER-37, 0.2 µg/mL). B–D show the net histamine release, i.e., stimulated release–spontaneous release (in % of total histamine). MCs from the same preparation are shown as interconnected dots (n = 12–15), *** *p* < 0.001, ns, not significant.

In skin-derived MCs, TSLP alone had likewise no impact on histamine release, i.e., did neither degranulate MCs on its own nor inhibit spontaneous histamine liberation (Figure 1A). Conversely, cells exposed to TSLP for 30 min prior to stimulation by the canonical MRGPRX2 ligands c48/80 (exogenous) or SP (endogenous) showed effective potentiation of histamine liberation over the secretagogues alone (Figure 1B,C). The effect was similar across multiple MC cultures, resulting in significant differences between pretreatments. Cells primed with TSLP and subsequently exposed to FcεRI aggregation showed an increase in some cultures only, while due to high variability across MC cultures, significance was not reached and no overall increase in FcεRI-triggered degranulation therefore detected (Figure 1D). The effect of TSLP on MC degranulation was not due to enhanced expression of MRGPRX2 at the cell surface, which remained comparable after TSLP (Supplementary Materials Figure S1A,B). This suggested that it was the signaling events initiated by the TSLP/TSLPR axis that synergized with the cascade elicited by MRGPRX2 to evoke degranulation.

Collectively, TSLP selectively promotes MRGPRX2-driven degranulation of human skin MCs.

### 3.2. TSLP Modestly Prolongs CD107a Exteriorization Following MRGPRX2-Triggering

Degranulation is accompanied by transient detection of granule markers at the cell surface as a result of granule-plasma membrane fusion, whereby CD107a best distinguishes activated from non-activated MCs, which are visible as two clearly separated populations [5,55] and it was also recently used to establish a pre-diagnostic MC activation test [61].

**Figure 2:**
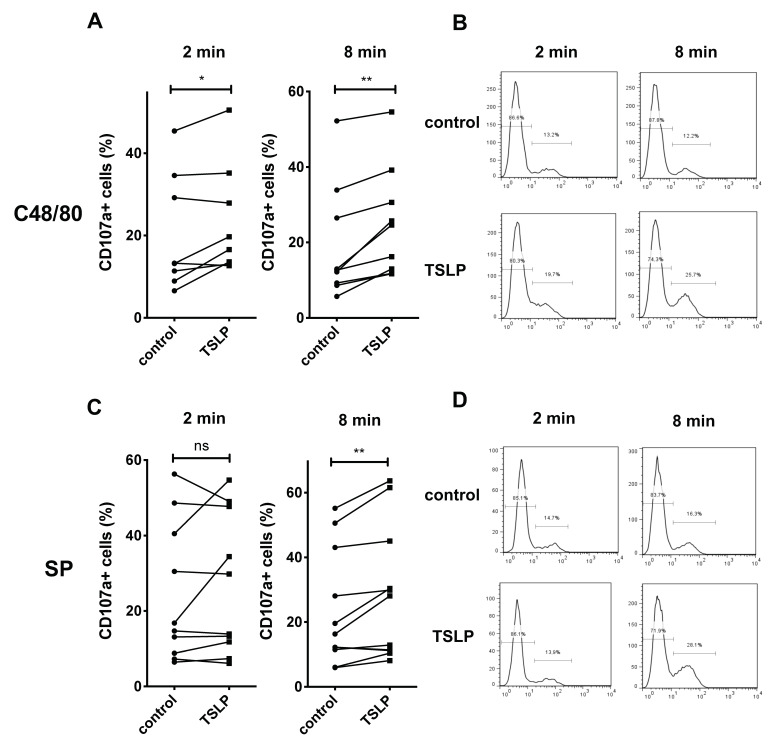
TSLP modestly increases the proportion of CD107a+ cells following MRGPRX2 triggering. MCs were pre-treated with or without TSLP (7.5 ng/mL), then stimulated with c48/80 (10 µg/mL) or SP (30 µM) for 2 min and 8 min, respectively; CD107a cell surface expression was determined by flow-cytometry. MCs from the same preparation are shown as interconnected dots (**A**,**C**). (**A**) Stimulation by c48/80, cumulative data. (**B**) Representative histogram of A. (**C**) Stimulation by SP, cumulative data. (**D**) Representative histogram of C. The data are from 8—11 independent experiments. * *p* < 0.05, ** *p* < 0.01, ns, not significant.

Prior treatment with TSLP led to slightly increased expression of CD107a following SP or c48/80 stimulation in a time-dependent fashion (Figure 2). The effect was stronger after 8 than 2 min, implying a lengthened opening of the granule, i.e., less efficient reuptake of CD107a in the presence of TSLP (Figure 2). Note that the response to MRGPRX2 ligands is very rapid, with CD107a exteriorization observable at 2 min and gradual reuptake within ≈ 15 to 30 min [5]. We also assessed the influence of TSLP on CD107a exteriorization upon FcεRI aggregation. This process is much slower than upon MRGPRX2 ligation in line with the different degranulation kinetics [24]. In two time-course series, TSLP had little effect on CD107a+ cells at any time point tested, i.e., 15, 30, or 60 min (Figure S2). The same was found when a larger number of MC preparations was assessed at one time point (30 min), though there was a slight tendency to reduced CD107a-positivity elicited via FcεRI in samples showing robust CD107a exteriorization (Figure S3). Treatment with TSLP alone had likewise no effect on the low proportion of cells spontaneously expressing CD107a (Figure S3).

**Figure 3:**
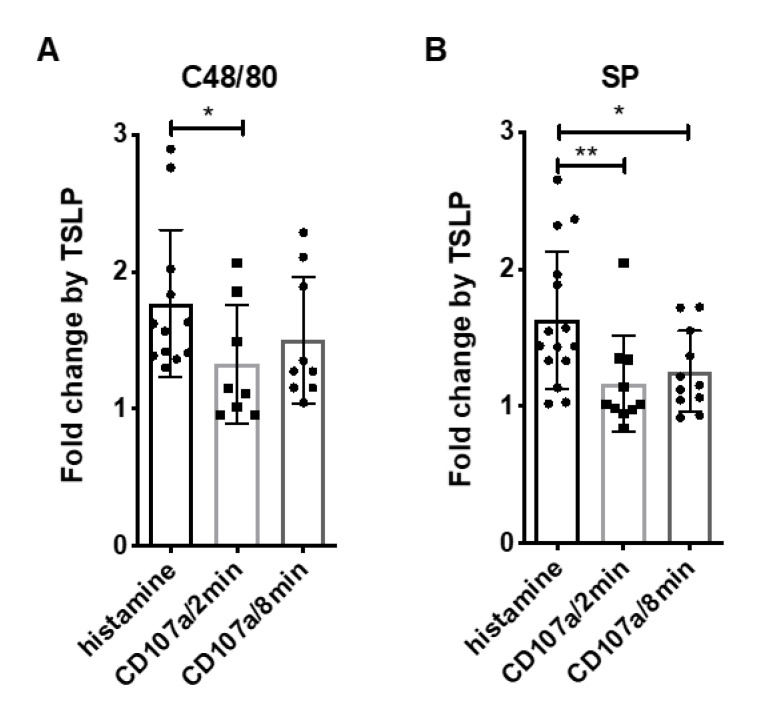
TSLP priming has greater impact on MRGPRX2-elicited histamine release than on CD107a exteriorization. The priming efficiency of TSLP (7.5 ng/mL) was compared between histamine release (according to Figure 1) and CD107a exteriorization (according to Figure 2). (**A**) c48/80 (10 µg/mL), (**B**) SP (30 µM). The data are presented as fold change (FC) by TSLP against control. Mean ± SD of n = 8–15 independent experiments. * *p* < 0.05, ** *p* < 0.01.

Since the impact of TSLP priming seemed more robust at the level of histamine release compared to CD107a-positive cells, we quantified the respective effect of TSLP as a fold-change ratio and indeed found a significant difference between outcomes (Figure 3). This underlines that the two measures grasp distinct aspects of the degranulation machinery that do not necessarily match, even though they often correlate. As expected, no difference was found for the FcεRI-route (Figure S4).

Collectively, TSLP has a positive, yet modest impact on the percentage of responding MCs, while prolonged granule opening may be the reason for the more efficient histamine release.

### 3.3. JNK Modestly Contributes to the Degranulation of Skin MCs in the Absence of TSLP, While STAT5 Has no Effect

We recently elaborated that among several signaling cascades, TSLP selectively elicits STAT5 and JNK activation in skin MCs [44]. This was verified in the present study. As in the previous report [44], immunoblots revealed some phosphorylated (p-)STAT5 already at baseline, which was further enhanced by TSLP (Figure S5A,B). We also found TSLP-elicited shifts in pSTAT5 and pJNK signals by FACS (Figure S5C,D); FACS and immunoblot findings match, as reported previously [44].

The focus of the current study was to dissect which of the cascades, if any, was required for TSLP-supported degranulation. Prior to examining this aspect, it was mandatory to realize whether the signaling components participate in granule discharge of skin MCs in the absence of TSLP.

**Figure 4:**
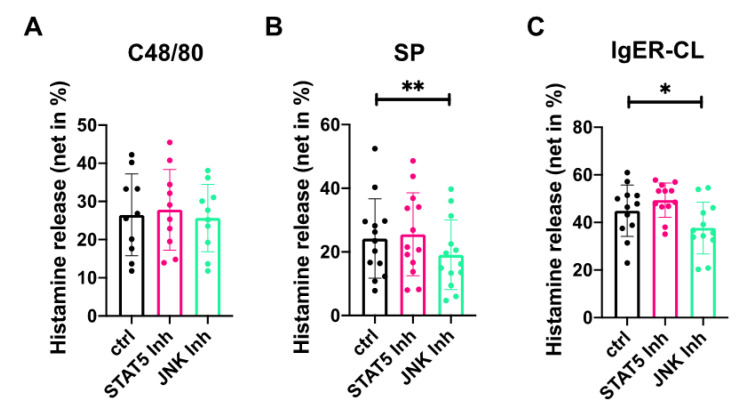
Inhibition of JNK slightly interferes with degranulation triggered by SP or FcεRI aggregation, while STAT5 perturbation has no effect. MCs were pretreated with the JNK inhibitor SP600125 (5 µM), the STAT5 inhibitor Pimozide (5 µM) or no inhibitor (buffer control) for 15 min, then stimulated with (**A**) c48/80 (10 µg/mL); (**B**) SP (30 µM); (**C**) IgER-CL (AER-37, 0.2 µg/mL) for 30 min. The net histamine release is given as mean ± SD of 10-13 independent experiments. * *p* < 0.05, ** *p* < 0.01.

The STAT5-inhibitor Pimozide had no effect on either FcεRI- or MRGPRX2-triggered degranulation (Figure 4). Conversely, while the JNK inhibitor SP600125 did not significantly modify c48/80-triggered degranulation, it slightly (but significantly) inhibited SP-elicited and FcεRI-triggered histamine liberation by a mean 21% and 16%, respectively (Figure 4).

### 3.4. The Promotion of MRGPRX2 Function by TSLP Is STAT5-Dependent with Further Contribution from JNK

We next explored whether JNK and STAT5 are implicated in TSLP’s promotion of exocytosis. Using a three-component setting (i.e., ±inhibitor, ±TSLP, ±stimulus), we found that this was indeed the case.

**Figure 5:**
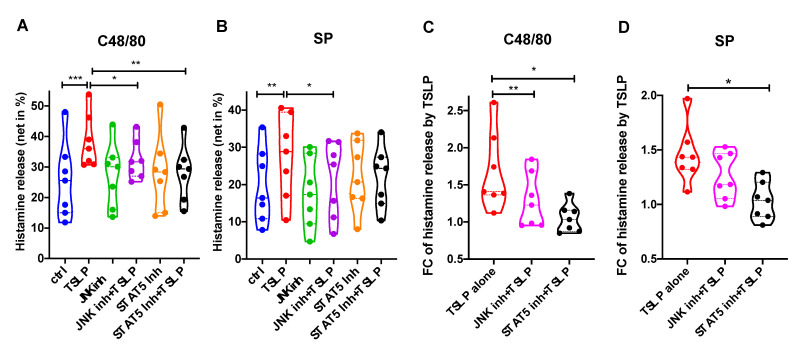
TSLP support of MRGPRX2-triggered degranulation depends on STAT5 and JNK. MCs were pretreated with the JNK inhibitor SP600125 (5 µM), the STAT5 inhibitor Pimozide (5 µM) or no inhibitor (buffer control) for 15 min, then stimulated with TSLP (7.5 ng/mL) for 30 min. (**A**,**B**) Histamine release was assessed after further 30 min with c48/80 (10 µg/mL) or SP (30 µM), and the net release calculated. (**C**,**D**) Fold change (FC) of histamine release in the presence of TSLP, calculated as ratio (with TSLP)/(without TSLP). The data are from 7 independent experiments. * *p* < 0.05, ** *p* < 0.01, *** *p* < 0.001. In A and B, the relevant comparisons (groups: control, JNK inhibitor + TSLP, STAT5 inhibitor + TSLP) were made against the “TSLP” group (red violin); in **C** and **D**, inhibitor groups were compared with “TSLP alone” (red violin).

In the overall view of all groups (Figure 5A,B), TSLP-mediated enhancement (red violin) was countered by both JNK-inhibitor (purple) and STAT5-inhibitor (black) to a similar extent (though the STAT5-inhibitor did not quite reach significance in the SP-panel owing to the ANOVA setting). However, since the JNK-inhibitor had a suppressive effect on SP-triggered degranulation in the absence of TSLP (as elaborated in the previous paragraph, Figure 4), it was important to account for this confounding effect and compare ratios of the three inhibitor conditions (null, JNK, STAT5) in the presence/absence of TSLP. In doing so, we found that the STAT5-inhibitor was able to completely reverse the effect of TSLP (nearly down to a ratio of one, signifying no remaining effect of TSLP at all) (Figure 5C,D). Interference with JNK likewise impeded TSLP from exerting its pro-secretory effect, yet the mean ratio was still well above one, i.e., there was no complete reversal (Figure 5C,D).

### 3.5. STAT5 Is Indispensable for TSLP-Promoted Degranulation—Verification by RNA Interference

**Figure 6:**
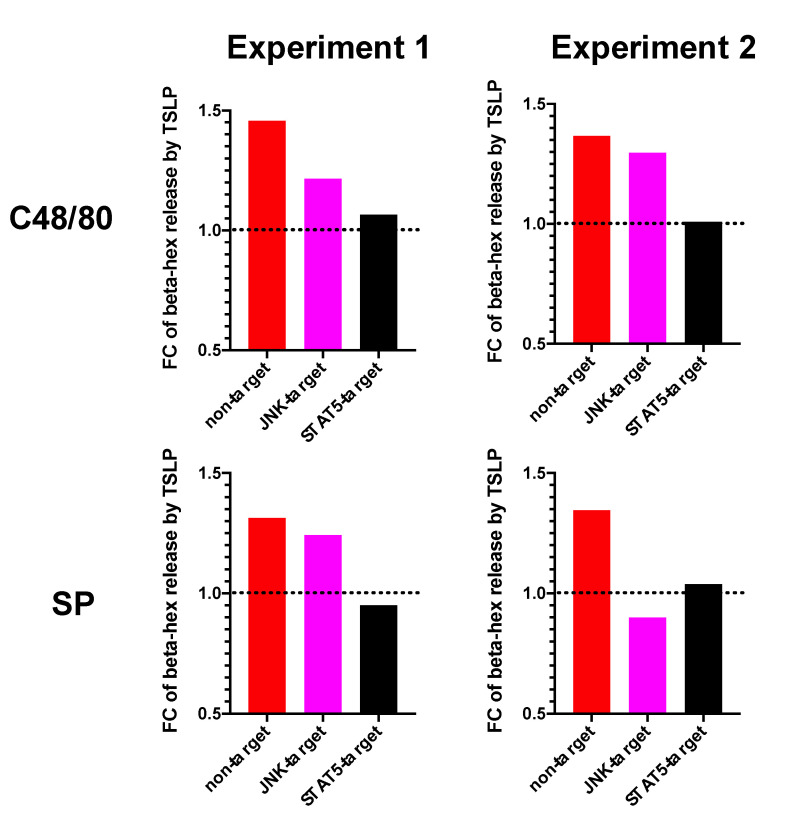
TSLP acts via STAT5 to increase MRGPRX2-mediated degranulation—verification by RNAi-mediated knockdown. MCs were subjected to RNA interference as described in Methods for 2 d, after which time cells were pre-treated or not with TSLP (7.5 ng/mL) prior to being stimulated by c48/80 (10 µg/mL) or SP (30 µM). Beta-hex(osaminidase) release was assessed after 30 min, spontaneous release was assessed analogously, and the net release calculated. Results from two separate experiments (MC cultures) are depicted as ratios “with TSLP/without TSLP” for the different siRNAs (calculated as in Figure 5C,D). The net beta-hex release of these experiments (analogous to Figure 5A,B) can be found as Figure S6.

Exploiting our recently established Accell^®^ mediated RNAi protocol [5,28,54], which also efficiently ablates STAT5- and JNK upon administration of siRNAs targeting these two components [44], we finally sought to validate the results from pharmacological inhibition with another technique. Interestingly, the STAT5-knockdown completely reversed the effect of TSLP, again moving the “with TSLP/without TSLP” ratio to 1, signifying no remaining effect of TSLP (Figure 6). This was found in both independent experiments and for the two MRGPRX2 agonists alike. Some involvement of JNK appeared likely, because the ratio was likewise (slightly or prominently) reduced in all four combinations, yet the effects were smaller. Taken together, the outcomes obtained with the two methods, i.e., inhibitors and RNAi, were perfectly in accord regarding both STAT5 and JNK.

We conclude that STAT5 is absolutely essential for TSLP’s supportive effect on pseudo-allergic degranulation, while JNK has a further contributory role.

## 4. Discussion

MCs adjust their responsiveness to secretory stimuli in dependence of the tissue microenvironment, whereby modulating factors like cytokines can critically influence their secretory competence without acting as secretagogues on their own. In doing so, extracellular stimuli can modify the manifestation and course of MC-dependent diseases by diminishing or increasing the concentration thresholds of triggering factors like allergens or drugs [49,62,63]. However, interconnections between degranulation-competent receptors and degranulation-incompetent modulatory factors are incompletely understood.

Degranulation can be efficiently elicited via two major routes in skin MCs, i.e., FcεRI (allergic) and MRGPRX2 (pseudo-allergic/neurogenic). The former involves the high affinity IgE receptor on the MC surface, antigen-specific IgE bound to it, and the antigen (allergen) which cross-links FcεRI to start a cascade which culminates in exocytosis [1,2,25,64]. Despite the plethora of ligands that trigger pseudo-allergic responses, the culprit receptor was discovered fairly recently as MRGPRX2 (or Mrgprb2 in the mouse) [3,7]. Agonists encompass not only multiple cationic drugs, but also antimicrobial and neuropeptides like Substance P (SP), making it a universally operating receptor of MCTC-type MCs [4,6]. MRGPRX2’s importance as a central signaling unit of this MC type has been widely recognized by the MC community [10,11,12,13,16,65,66,67]. As research into MRGPRX2 began only recently, less is known about its modulation by autocrine and paracrine factors compared to FcεRI.

Notwithstanding, several themes have started to emerge. In particular, MRGPRX2-triggered degranulation can be negatively affected by cues typically supportive of the lineage. Specifically, the dominant MC growth factor SCF interferes with the pseudo-allergic route, while simultaneously strengthening allergic stimulation [18,19]. In addition, IL-4 and (prolonged) IL-33 dampen MRGPRX2 functionality [19,28]. Accordingly, MRGPRX2 function becomes gradually diminished in the SCF-rich conditions of in vitro MC culture, while the FcεRI-route is simultaneously boosted [19,51,52]. It is still a mystery how MCs in the skin (and directly ex vivo) preserve their potent responsiveness to MRGPRX2 agonists but are unable to fully maintain this function in culture [19], leaving open the question of how MRGPRX2 is supported by the skin environment, and whether mediators exist that favor MRGPRX2- over FcεRI function.

TSLP, mostly expressed by epithelial cells, is considered a Th2-promoting master switch implicated in Th2-dominated pathologies in the skin, lung and other organs, and is therefore a focus in allergy-related research [35,39,68,69,70,71,72]. Considering this intimate relation and the assumption of MRGPRX2 being a key structure in AD [9], we hypothesized that TSLP may constitute one of the factors promoting the MRGPRX2 route, and found that this is indeed the case.

MC modulating activities on maturation markers, production of cytokines and survival have previously been ascribed to TSLP [44,45,56,57,73,74,75]. For example, the cytokine was shown to synergize with pro-inflammatory mediators to enhance MC production of Th2-cytokines in the absence of FcεRI aggregation [45]. To our knowledge, no study has yet demonstrated that TSLP can assist in granule discharge, however, which may be viewed as the most selective MC function. In fact, focusing on FcεRI-mediated activation, Joulia et al. found no effect of TSLP on MC secretory competence in peripheral blood derived MCs, while the simultaneously investigated IL-33 efficiently augmented this function [60]. The lacking effect of TSLP on allergic stimulation was reproduced in our study (Figure 1D for histamine release, Figures S2 and S3 for CD107a upregulation after IgER-CL). The group did not study MC activation triggered via the alternative, pseudo-allergic route. Rönnberg et al. reported that TSLP did not induce histamine release from either CBMCs or the ROSA MC line [75], a finding likewise duplicated in our study for skin-derived MCs (Figure 1A for spontaneous histamine release, Figures S2 and S3 for CD107a expression). The discovery that TSLPR and MRGPRX2 synergize when co-activated is therefore intriguing, especially since this is found for the natural producers of MRGPRX2, and since TSLP modifies mediator secretion in a way that differentiates between the MRGPRX2- and the FcεRI-driven process, favoring the former.

The sensitivity of skin MCs towards TSLP can be linked to the abundant expression of the TSLP receptor in these cells [17,42,44]. The cytokine can activate distinct cascades depending on cell type, but activation of STAT5 represents the canonical and best documented pathway in most TSLPR expressing cells [76,77,78,79]. We recently elaborated the mechanistic underpinnings of TSLP action in skin MCs, revealing activation of STAT5 and JNK, but not of STAT3, p38 or ERK1/2 [44]. STAT5 and JNK activation were reproduced in the current study (Figure S5). Complementation of STAT5 by JNK was rather unexpected, because JNK had only occasionally been found downstream of the TSLP/TSLPR axis [80]. Nonetheless, we showed that not only STAT5 but also JNK is a crucial component of apoptosis resistance through activation of Bcl-xl expression, which together with the parallel STAT5/Mcl-1 axis leads to survival promotion [44].

We therefore asked whether STAT5, JNK, both or none form part of the apparatus, by which TSLP potentiates MRGPRX2-dependent degranulation. To be able to judge, we first needed to clarify whether JNK and STAT5 are involved in MRGPRX2-evoked degranulation in the absence of TSLP. STAT5 perturbation had no impact on histamine liberation whatsoever, and this applied not only to the MRGPRX2- but also to the canonical IgER-route studied in parallel for comparison (Figure 4). Dependence on STAT5 may differ in other MC subsets, however. In fact, STAT5 tyrosine phosphorylation was elicited downstream of FcεRI-aggregation in murine bone-marrow derived cultured MCs (BMcMCs) and contributed to FcεRI-driven histamine discharge in these cells [81,82]. Moreover, though signaling events downstream of MRGPRX2 are still rudimentarily understood, inhibition of STAT5 by Pimozide (i.e., the same inhibitor as used in this study) did (modestly) interfere with degranulation triggered by MRGPRX2 agonists in the LAD2 cell line [83]. This further substantiates that MC heterogeneity precludes extrapolations from one MC type to another, likely due in part to the varying levels and ratios of crucial signaling components across MC subsets. Importantly, in skin-derived MCs, the pathophysiological responders to MRGPRX2 ligands, STAT5 does not appear to make a meaningful contribution to granule discharge triggered by either the allergic or the pseudo-allergic/neurogenic pathway unless TSLP is present (as detailed below).

JNK is a well-established event in the signaling cascade downstream of FcεRI that can be activated by multiple upstream kinases, including MKK7, PI3K or PKC [84,85]. So far, JNK function in MCs has been chiefly associated with proliferation and survival [86,87]. In contrast, a connection with degranulation is limited at best, though one study did show involvement in degranulation triggered via an FcγR-dependent route in the mouse [88]. JNK can influence the production of cytokines like IL-31 and TNF-α elicited by typical MRGPRX2 agonists in LAD2, progenitor-derived MCs and rat MCs, however [89,90]. In another report, JNK was found to partake in cytokine stimulation by SP in LAD2 cells (secondary to Go knockdown), but was explicitly not involved in degranulation [91].

Here we found a slight, yet significant effect of JNK perturbation on degranulation triggered by SP (as well as FcεRI), yet not by c48/80 (Figure 4). In fact, despite utilizing the same receptor, SP and c48/80 show several differences when inspected at finer resolution. For example, SP is less potent at inducing MRGPRX2 internalization compared to c48/80 [5]. In a previous study we found that interference with JNK had no significant effect on MC degranulation with a lower number of independent experiments [28]. This underlines that only with highly powered experiments (n > 10), an impact of JNK can be revealed in a statistical fashion, highlighting a rather slight and/or variable effect, which may stem, at least in part, from MC divergence across donors [18,49,50,62]. Together, JNK seems to (moderately) contribute to FcεRI, and especially to SP-triggered histamine release in skin-derived MCs even in the absence of TSLP.

In contrast to the standard situation, STAT5 activity was indispensable to the pro-secretory function of TSLP, which was nullified by Pimozide. JNK supported STAT5, although its inhibition had a less complete effect than that of STAT5. This was discernible in the ratio-centered view, whereby TSLP still had a supportive (yet reduced) effect under JNK inhibition, while this effect was virtually absent upon interference with STAT5. The indispensable nature of STAT5 was verified following its RNAi-mediated knockdown. In this setting TSLP was unable to increase degranulation in both independent experiments and to both stimuli (c48/80 and SP) (Figure 6). The impact of JNK could not be fully ascertained in the RNAi experiments due to the less potent impact of its elimination, but promotion by TSLP was reduced rather than increased in the four experimental settings using JNK-RNAi (two stimuli used on two MC cultures, Figure 6). This result was perfectly in accord with the result from pharmacological inhibition (magenta versus red in Figure 5 and Figure 6, respectively).

In summary, while STAT5 is the dominant entity, joint action of STAT5 and JNK is likely required to optimally orchestrate the heightened secretory competence bestowed by TSLP. Synchronized activation of STAT5 and JNK may thus mediate different TSLP outcomes in skin MCs, including survival protection [44] and MRGPRX2-elicited granule discharge (this study).

MRGPRX2 has moved to the center of attention not least because of the large and still increasing number of secretagogues activating the receptor, of which many can be linked to symptoms or diseases. These include injection-site hypersensitivity, red man syndrome, and anaphylactic reactions to an array of drugs (e.g., muscle relaxants, opiates, and antibiotics) [4,6,8,13,16,92]. In addition to these acute responses, MRGPRX2 also contributes to complex chronic diseases of the skin, including chronic idiopathic urticaria (CIU) and AD [4,6,9]. Regarding CIU, it was reported that urticaria patients are characterized by overexpression of MRGPRX2 [20], while a newer publication confirmed this by also showing enhanced skin reactivity of patients to MRGPRX2 drug ligands [93]. Importantly, TSLP-positive cells are also increased in CIU skin [94].

As mentioned, aberrant TSLP activity is closely tied to AD, and tezepelumab (anti-TSLP antibody) is in clinical trials for this condition [37]. Of importance, TSLP-mediated AD is independent of adaptive immunity in mouse models but rather associated with heightened numbers of innate immune cells, including MCs, as elaborated by several groups harnessing T cell/B cell deficient mice along with skin-restricted TSLP overexpression [70,71,72]. Studies showing that TSLP protects against MC death [44,73] indicate that TSLP affords direct support to MCs in these models. The current study provides evidence that TSLP additionally promotes activation via the MRGPRX2-route. This is notable because despite the wide appreciation of MCs’ crucial part in AD pathology, it is still unresolved how exactly the cells are activated in skin lesions to initiate or perpetuate the disease [48], yet evidence is growing that MRGPRX2 may constitute a missing link at least in selected AD endotypes [9]. This is further underlined by the bidirectional crosstalk between MCs and sensory neurons in AD pathology [95], while MRGPRX2 happens to be the major MC-expressed receptor for neuropeptides like SP, vasoactive intestinal peptide (VIP), somatostatin, cortistatin, and Pituitary adenylate cyclase-activating peptide (PACAP) [3,6]. Our study further extends the connection between AD, TSLP and MCs. It is also remarkable in this context that not only TSLP is enhanced in AD lesions, but so is phosphorylated STAT5 in lesional MCs [96].

The relevance of overexpressed TSLP and deregulated MRGPRX2 activity in skin pathologies indicates that their synergy may have important ramifications for understanding and treatment of skin disorders, but also host-defenses organized by MRGPRX2 on the other end of the spectrum. In fact, evidence is accumulating that MRGPRX2/Mrgprb2 can mobilize anti-bacterial mechanisms, e.g., after exogenous activation in the context of Staphylococcus aureus induced dermonecrotic lesions [97] or by responding to Quorum-sensing molecules secreted by bacteria to signal population density [98].

It remains to be elucidated whether newly synthesized mediators like cytokines can be elicited via MRGPRX2 in human skin MCs, i.e., whether this MC type behaves rather like cells of the leukemic LAD2 line (efficient production of cytokines with MRGPRX2 agonists) or rather like (peripheral-blood derived) primary MCs (cytokine production inefficient or undetectable) [24,99]. Differences across MC subsets regarding MRGPRX2-triggered cytokine generation were recently summarized [4]. We are currently exploring this aspect for human cutaneous MCs, and once established, future studies will have to assess whether TSLP can influence the other key MC function in cells from human dermis.

As of now, TSLP may be provisionally assumed to produce a hyperactive phenotype with selectivity for MRGPRX2, as has been suggested for aberrant KIT activity (due to SCF overexpression or the D816V mutation) in connection with allergen-dependent activation [100]. In conclusion, we show that the Th2-associated cytokine TSLP selectively promotes MRGPRX2-dependent degranulation of skin MCs, being arguably the first cytokine to favor pseudo-allergic over allergic degranulation. Mechanistically, the priming effect depends on STAT5, supplemented by JNK activity. The relevance of TSLP, MCs and MRGPRX2 to pruritis and atopic pathology but also host defenses indicates wide repercussions of the identified connection.

## Data Availability

No datasets were generated or analyzed during this study.

## Supplementary Materials

The following are available online at https://www.mdpi.com/2073-4409/10/1/102/s1, Figure S1: TSLP priming does not change MRGPRX2 surface expression. Figure S2: TSLP priming has no impact on the time course of CD107a exteriorization after FcεRI aggregation. Figure S3: No effect of TSLP on the spontaneous or FcεRI-elicited proportion of CD107+ cells in multiple MC preparations. Figure S4: Comparison between FcεRI-triggered histamine release and CD107a exteriorization in the presence of TSLP. Figure S5: TSLP elicits phosphorylation of STAT5 in skin MCs. Figure S6: STAT5 is essential for TSLP to support MRGPRX2-mediated degranulation—insights from RNAi-mediated knockdown.

## References

[1] Metcalfe D.D., Peavy R.D., Gilfillan A.M. (2009). Mechanisms of mast cell signaling in anaphylaxis. J. Allergy Clin. Immunol..

[2] Galli S.J., Tsai M. (2012). IgE and mast cells in allergic disease. Nat. Med..

[3] McNeil B.D., Pundir P., Meeker S., Han L., Undem B.J., Kulka M., Dong X. (2015). Identification of a mast-cell-specific receptor crucial for pseudo-allergic drug reactions. Nature.

[4] Babina M. (2020). The pseudo-allergic/neurogenic route of mast cell activation via MRGPRX2: Discovery, functional programs, regulation, relevance to disease, and relation with allergic stimulation. Itch.

[5] Babina M., Wang Z., Roy S., Guhl S., Franke K., Artuc M., Ali H., Zuberbier T. (2020). MRGPRX2 is the codeine receptor of human skin mast cells: Desensitization via β-arrestin and lack of correlation with the FcεRI pathway. J. Investig. Dermatol..

[6] Kühn H., Kolkhir P., Babina M., Düll M., Frischbutter S., Fok J.S., Jiao Q., Metz M., Scheffel J., Wolf K. (2020). Mas-related G protein–coupled receptor X2 and its activators in dermatologic allergies. J. Allergy Clin. Immunol..

[7] Tatemoto K., Nozaki Y., Tsuda R., Konno S., Tomura K., Furuno M., Ogasawara H., Edamura K., Takagi H., Iwamura H. (2006). Immunoglobulin E-independent activation of mast cell is mediated by Mrg receptors. Biochem. Biophys. Res. Commun..

[8] Lansu K., Karpiak J., Liu J., Huang X.P., McCorvy J.D., Kroeze W.K., Che T., Nagase H., Carroll F.I., Jin J. (2017). In silico design of novel probes for the atypical opioid receptor MRGPRX2. Nat. Chem. Biol..

[9] Wang Z., Babina M. (2020). MRGPRX2 signals its importance in cutaneous mast cell biology: Does MRGPRX2 connect mast cells and atopic dermatitis?. Exp. Derm..

[10] Kim H.S., Kawakami Y., Kasakura K., Kawakami T. (2020). Recent advances in mast cell activation and regulation. F1000Res.

[11] Olivera A., Beaven M.A., Metcalfe D.D. (2018). Mast cells signal their importance in health and disease. J. Allergy Clin. Immunol..

[12] Lyons D.O., Pullen N.A. (2020). Beyond IgE: Alternative Mast Cell Activation Across Different Disease States. Int. J. Mol. Sci..

[13] Ali H. (2017). Emerging Roles for MAS-Related G Protein-Coupled Receptor-X2 in Host Defense Peptide, Opioid, and Neuropeptide-Mediated Inflammatory Reactions. Adv. Immunol..

[14] Subramanian H., Gupta K., Ali H. (2016). Roles of Mas-related G protein-coupled receptor X2 on mast cell-mediated host defense, pseudoallergic drug reactions, and chronic inflammatory diseases. J. Allergy Clin. Immunol..

[15] Chompunud Na Ayudhya C., Roy S., Alkanfari I., Ganguly A., Ali H. (2019). Identification of Gain and Loss of Function Missense Variants in MRGPRX2’s Transmembrane and Intracellular Domains for Mast Cell Activation by Substance P. Int. J. Mol. Sci..

[16] Porebski G., Kwiecien K., Pawica M., Kwitniewski M. (2018). Mas-Related G Protein-Coupled Receptor-X2 (MRGPRX2) in Drug Hypersensitivity Reactions. Front. Immunol..

[17] Motakis E., Guhl S., Ishizu Y., Itoh M., Kawaji H., de Hoon M., Lassmann T., Carninci P., Hayashizaki Y., Zuberbier T. (2014). Redefinition of the human mast cell transcriptome by deep-CAGE sequencing. Blood.

[18] Babina M., Guhl S., Artuc M., Zuberbier T. (2018). Allergic FcepsilonRI- and pseudo-allergic MRGPRX2-triggered mast cell activation routes are independent and inversely regulated by SCF. Allergy.

[19] Babina M., Wang Z., Artuc M., Guhl S., Zuberbier T. (2018). MRGPRX2 is negatively targeted by SCF and IL-4 to diminish pseudo-allergic stimulation of skin mast cells in culture. Exp. Derm..

[20] Fujisawa D., Kashiwakura J., Kita H., Kikukawa Y., Fujitani Y., Sasaki-Sakamoto T., Kuroda K., Nunomura S., Hayama K., Terui T. (2014). Expression of Mas-related gene X2 on mast cells is upregulated in the skin of patients with severe chronic urticaria. J. Allergy Clin. Immunol..

[21] Bischoff S.C., Schwengberg S., Lorentz A., Manns M.P., Bektas H., Sann H., Levi-Schaffer F., Shanahan F., Schemann M. (2004). Substance P and other neuropeptides do not induce mediator release in isolated human intestinal mast cells. Neurogastroenterol. Motil..

[22] Lowman M.A., Rees P.H., Benyon R.C., Church M.K. (1988). Human mast cell heterogeneity: Histamine release from mast cells dispersed from skin, lung, adenoids, tonsils, and colon in response to IgE-dependent and nonimmunologic stimuli. J. Allergy Clin. Immunol..

[23] Tharp M.D., Kagey-Sobotka A., Fox C.C., Marone G., Lichtenstein L.M., Sullivan T.J. (1987). Functional heterogeneity of human mast cells from different anatomic sites: In vitro responses to morphine sulfate. J. Allergy Clin. Immunol..

[24] Gaudenzio N., Sibilano R., Marichal T., Starkl P., Reber L.L., Cenac N., McNeil B.D., Dong X., Hernandez J.D., Sagi-Eisenberg R. (2016). Different activation signals induce distinct mast cell degranulation strategies. J. Clin. Investig..

[25] Gilfillan A.M., Tkaczyk C. (2006). Integrated signalling pathways for mast-cell activation. Nat. Rev. Immunol..

[26] Gilfillan A.M., Beaven M.A. (2011). Regulation of mast cell responses in health and disease. Crit. Rev. Immunol..

[27] Metcalfe D.D. (2008). Mast cells and mastocytosis. Blood.

[28] Wang Z., Guhl S., Franke K., Artuc M., Zuberbier T., Babina M. (2019). IL-33 and MRGPRX2-Triggered Activation of Human Skin Mast Cells-Elimination of Receptor Expression on Chronic Exposure, but Reinforced Degranulation on Acute Priming. Cells.

[29] Babina M., Guhl S., Motakis E., Artuc M., Hazzan T., Worm M., Forrest A.R., Zuberbier T. (2015). Retinoic acid potentiates inflammatory cytokines in human mast cells: Identification of mast cells as prominent constituents of the skin retinoid network. Mol. Cell Endocrinol..

[30] Babina M., Artuc M., Guhl S., Zuberbier T. (2017). Retinoic Acid Negatively Impacts Proliferation and MCTC Specific Attributes of Human Skin Derived Mast Cells, but Reinforces Allergic Stimulability. Int. J. Mol. Sci..

[31] Noti M., Kim B.S., Siracusa M.C., Rak G.D., Kubo M., Moghaddam A.E., Sattentau Q.A., Comeau M.R., Spergel J.M., Artis D. (2014). Exposure to food allergens through inflamed skin promotes intestinal food allergy through the thymic stromal lymphopoietin-basophil axis. J. Allergy Clin. Immunol..

[32] O’Shea K.M., Aceves S.S., Dellon E.S., Gupta S.K., Spergel J.M., Furuta G.T., Rothenberg M.E. (2018). Pathophysiology of Eosinophilic Esophagitis. Gastroenterology.

[33] Locksley R.M. (2010). Asthma and allergic inflammation. Cell.

[34] Spergel J.M., Paller A.S. (2003). Atopic dermatitis and the atopic march. J. Allergy Clin. Immunol..

[35] Soumelis V., Reche P.A., Kanzler H., Yuan W., Edward G., Homey B., Gilliet M., Ho S., Antonenko S., Lauerma A. (2002). Human epithelial cells trigger dendritic cell mediated allergic inflammation by producing TSLP. Nat. Immunol..

[36] Ziegler S.F., Artis D. (2010). Sensing the outside world: TSLP regulates barrier immunity. Nat. Immunol..

[37] Simpson E.L., Parnes J.R., She D., Crouch S., Rees W., Mo M., van der Merwe R. (2019). Tezepelumab, an anti-thymic stromal lymphopoietin monoclonal antibody, in the treatment of moderate to severe atopic dermatitis: A randomized phase 2a clinical trial. J. Am. Acad. Derm..

[38] Corren J., Parnes J.R., Wang L., Mo M., Roseti S.L., Griffiths J.M., van der Merwe R. (2017). Tezepelumab in Adults with Uncontrolled Asthma. N. Engl. J. Med..

[39] Ziegler S.F. (2012). Thymic stromal lymphopoietin and allergic disease. J. Allergy Clin. Immunol..

[40] Salazar F., Ghaemmaghami A.M. (2013). Allergen recognition by innate immune cells: Critical role of dendritic and epithelial cells. Front. Immunol..

[41] Milovanovic M., Volarevic V., Radosavljevic G., Jovanovic I., Pejnovic N., Arsenijevic N., Lukic M.L. (2012). IL-33/ST2 axis in inflammation and immunopathology. Immunol. Res..

[42] Forrest A.R., Kawaji H., Rehli M., Baillie J.K., de Hoon M.J., Haberle V., Lassmann T., Kulakovskiy I.V., Lizio M., Itoh M. (2014). A promoter-level mammalian expression atlas. Nature.

[43] Noguchi S., Arakawa T., Fukuda S., Furuno M., Hasegawa A., Hori F., Ishikawa-Kato S., Kaida K., Kaiho A., Kanamori-Katayama M. (2017). FANTOM5 CAGE profiles of human and mouse samples. Sci. Data.

[44] Hazzan T., Eberle J., Worm M., Babina M. (2019). Thymic Stromal Lymphopoietin Interferes with the Apoptosis of Human Skin Mast Cells by a Dual Strategy Involving STAT5/Mcl-1 and JNK/Bcl-xL. Cells.

[45] Allakhverdi Z., Comeau M.R., Jessup H.K., Yoon B.R., Brewer A., Chartier S., Paquette N., Ziegler S.F., Sarfati M., Delespesse G. (2007). Thymic stromal lymphopoietin is released by human epithelial cells in response to microbes, trauma, or inflammation and potently activates mast cells. J. Exp. Med..

[46] Kumari V., Babina M., Hazzan T., Worm M. (2015). Thymic stromal lymphopoietin induction by skin irritation is independent of tumour necrosis factor-alpha, but supported by interleukin-1. Br. J. Derm..

[47] Redhu D., Franke K., Kumari V., Francuzik W., Babina M., Worm M. (2020). Thymic stromal lymphopoietin production induced by skin irritation results from concomitant activation of protease-activated receptor 2 and interleukin 1 pathways. Br. J. Derm..

[48] Kawakami T., Ando T., Kimura M., Wilson B.S., Kawakami Y. (2009). Mast cells in atopic dermatitis. Curr. Opin. Immunol..

[49] Babina M., Guhl S., Artuc M., Trivedi N.N., Zuberbier T. (2016). Phenotypic variability in human skin mast cells. Exp. Derm..

[50] Babina M., Wang Z., Franke K., Guhl S., Artuc M., Zuberbier T. (2019). Yin-Yang of IL-33 in Human Skin Mast Cells: Reduced Degranulation, but Augmented Histamine Synthesis through p38 Activation. J. Investig. Derm..

[51] Guhl S., Neou A., Artuc M., Zuberbier T., Babina M. (2014). Skin mast cells develop non-synchronized changes in typical lineage characteristics upon culture. Exp. Derm..

[52] Guhl S., Artuc M., Neou A., Babina M., Zuberbier T. (2011). Long-term cultured human skin mast cells are suitable for pharmacological studies of anti-allergic drugs due to high responsiveness to FcepsilonRI cross-linking. Biosci. Biotechnol. Biochem..

[53] Babina M., Guhl S., Starke A., Kirchhof L., Zuberbier T., Henz B.M. (2004). Comparative cytokine profile of human skin mast cells from two compartments--strong resemblance with monocytes at baseline but induction of IL-5 by IL-4 priming. J. Leukoc Biol..

[54] Hazzan T., Guhl S., Artuc M., Franke K., Worm M., Zuberbier T., Babina M. (2017). An efficient method for gene knock-down by RNA interference in human skin mast cells. Exp. Derm..

[55] Guhl S., Stefaniak R., Strathmann M., Babina M., Piazena H., Henz B.M., Zuberbier T. (2005). Bivalent effect of UV light on human skin mast cells-low-level mediator release at baseline but potent suppression upon mast cell triggering. J. Investig. Derm..

[56] Nagarkar D.R., Poposki J.A., Comeau M.R., Biyasheva A., Avila P.C., Schleimer R.P., Kato A. (2012). Airway epithelial cells activate TH2 cytokine production in mast cells through IL-1 and thymic stromal lymphopoietin. J. Allergy Clin. Immunol..

[57] Kaur D., Doe C., Woodman L., Heidi Wan W.Y., Sutcliffe A., Hollins F., Brightling C. (2012). Mast cell-airway smooth muscle crosstalk: The role of thymic stromal lymphopoietin. Chest.

[58] Allakhverdi Z., Comeau M.R., Armant M., Agrawal R., Woodfolk J.A., Sehmi R., Howie K.J., Gauvreau G.M., Delespesse G. (2013). Mast Cell-Activated Bone Marrow Mesenchymal Stromal Cells Regulate Proliferation and Lineage Commitment of CD34(+) Progenitor Cells. Front. Immunol..

[59] Buchheit K.M., Cahill K.N., Katz H.R., Murphy K.C., Feng C., Lee-Sarwar K., Lai J., Bhattacharyya N., Israel E., Boyce J.A. (2016). Thymic stromal lymphopoietin controls prostaglandin D2 generation in patients with aspirin-exacerbated respiratory disease. J. Allergy Clin. Immunol..

[60] Joulia R., L’Faqihi F.E., Valitutti S., Espinosa E. (2017). IL-33 fine tunes mast cell degranulation and chemokine production at the single-cell level. J. Allergy Clin. Immunol..

[61] Bahri R., Custovic A., Korosec P., Tsoumani M., Barron M., Wu J., Sayers R., Weimann A., Ruiz-Garcia M., Patel N. (2018). Mast cell activation test in the diagnosis of allergic disease and anaphylaxis. J. Allergy Clin. Immunol..

[62] Babina M., Guhl S., Artuc M., Zuberbier T. (2017). Skin mast cell phenotypes between two highly divergent cohorts—More pronounced variability within than between groups. Exp. Derm..

[63] Bousquet J., Anto J.M., Akdis M., Auffray C., Keil T., Momas I., Postma D.S., Valenta R., Wickman M., Cambon-Thomsen A. (2016). Paving the way of systems biology and precision medicine in allergic diseases: The MeDALL success story: Mechanisms of the Development of ALLergy; EU FP7-CP-IP; Project No: 261357; 2010–2015. Allergy.

[64] Lieberman P., Garvey L.H. (2016). Mast Cells and Anaphylaxis. Curr. Allergy Asthma Rep..

[65] Steinhoff M., Buddenkotte J., Lerner E.A. (2018). Role of mast cells and basophils in pruritus. Immunol. Rev..

[66] Espinosa-Riquer Z.P., Segura-Villalobos D., Ramirez-Moreno I.G., Perez Rodriguez M.J., Lamas M., Gonzalez-Espinosa C. (2020). Signal Transduction Pathways Activated by Innate Immunity in Mast Cells: Translating Sensing of Changes into Specific Responses. Cells.

[67] Redegeld F.A., Yu Y., Kumari S., Charles N., Blank U. (2018). Non-IgE mediated mast cell activation. Immunol. Rev..

[68] Cianferoni A., Spergel J. (2014). The importance of TSLP in allergic disease and its role as a potential therapeutic target. Expert Rev. Clin. Immunol..

[69] Volpe E., Pattarini L., Martinez-Cingolani C., Meller S., Donnadieu M.H., Bogiatzi S.I., Fernandez M.I., Touzot M., Bichet J.C., Reyal F. (2014). Thymic stromal lymphopoietin links keratinocytes and dendritic cell-derived IL-23 in patients with psoriasis. J. Allergy Clin. Immunol..

[70] Yoo J., Omori M., Gyarmati D., Zhou B., Aye T., Brewer A., Comeau M.R., Campbell D.J., Ziegler S.F. (2005). Spontaneous atopic dermatitis in mice expressing an inducible thymic stromal lymphopoietin transgene specifically in the skin. J. Exp. Med..

[71] Li M., Hener P., Zhang Z., Kato S., Metzger D., Chambon P. (2006). Topical vitamin D3 and low-calcemic analogs induce thymic stromal lymphopoietin in mouse keratinocytes and trigger an atopic dermatitis. Proc. Natl. Acad. Sci. USA.

[72] Briot A., Deraison C., Lacroix M., Bonnart C., Robin A., Besson C., Dubus P., Hovnanian A. (2009). Kallikrein 5 induces atopic dermatitis-like lesions through PAR2-mediated thymic stromal lymphopoietin expression in Netherton syndrome. J. Exp. Med..

[73] Han N.R., Oh H.A., Nam S.Y., Moon P.D., Kim D.W., Kim H.M., Jeong H.J. (2014). TSLP induces mast cell development and aggravates allergic reactions through the activation of MDM2 and STAT6. J. Investig. Derm..

[74] Saluja R., Zoltowska A., Ketelaar M.E., Nilsson G. (2016). IL-33 and Thymic Stromal Lymphopoietin in mast cell functions. Eur. J. Pharm..

[75] Ronnberg E., Ghaib A., Ceriol C., Enoksson M., Arock M., Safholm J., Ekoff M., Nilsson G. (2019). Divergent Effects of Acute and Prolonged Interleukin 33 Exposure on Mast Cell IgE-Mediated Functions. Front. Immunol..

[76] Scheeren F.A., van Lent A.U., Nagasawa M., Weijer K., Spits H., Legrand N., Blom B. (2010). Thymic stromal lymphopoietin induces early human B-cell proliferation and differentiation. Eur. J. Immunol..

[77] Kabata H., Moro K., Fukunaga K., Suzuki Y., Miyata J., Masaki K., Betsuyaku T., Koyasu S., Asano K. (2013). Thymic stromal lymphopoietin induces corticosteroid resistance in natural helper cells during airway inflammation. Nat. Commun..

[78] Bell B.D., Kitajima M., Larson R.P., Stoklasek T.A., Dang K., Sakamoto K., Wagner K.U., Kaplan D.H., Reizis B., Hennighausen L. (2013). The transcription factor STAT5 is critical in dendritic cells for the development of TH2 but not TH1 responses. Nat. Immunol..

[79] Yao W., Zhang Y., Jabeen R., Nguyen E.T., Wilkes D.S., Tepper R.S., Kaplan M.H., Zhou B. (2013). Interleukin-9 is required for allergic airway inflammation mediated by the cytokine TSLP. Immunity.

[80] Shan L., Redhu N.S., Saleh A., Halayko A.J., Chakir J., Gounni A.S. (2010). Thymic stromal lymphopoietin receptor-mediated IL-6 and CC/CXC chemokines expression in human airway smooth muscle cells: Role of MAPKs (ERK1/2, p38, and JNK) and STAT3 pathways. J. Immunol..

[81] Pullen N.A., Barnstein B.O., Falanga Y.T., Wang Z., Suzuki R., Tamang T.D., Khurana M.C., Harry E.A., Draber P., Bunting K.D. (2012). Novel mechanism for Fc{epsilon}RI-mediated signal transducer and activator of transcription 5 (STAT5) tyrosine phosphorylation and the selective influence of STAT5B over mast cell cytokine production. J. Biol. Chem..

[82] Barnstein B.O., Li G., Wang Z., Kennedy S., Chalfant C., Nakajima H., Bunting K.D., Ryan J.J. (2006). Stat5 expression is required for IgE-mediated mast cell function. J. Immunol..

[83] Hermans M.A.W., Schrijver B., van Holten-Neelen C., Gerth van Wijk R., van Hagen P.M., van Daele P.L.A., Dik W.A. (2018). The JAK1/JAK2- inhibitor ruxolitinib inhibits mast cell degranulation and cytokine release. Clin. Exp. Allergy.

[84] Kawakami Y., Hartman S.E., Holland P.M., Cooper J.A., Kawakami T. (1998). Multiple signaling pathways for the activation of JNK in mast cells: Involvement of Bruton’s tyrosine kinase, protein kinase C, and JNK kinases, SEK1 and MKK7. J. Immunol..

[85] Chayama K., Papst P.J., Garrington T.P., Pratt J.C., Ishizuka T., Webb S., Ganiatsas S., Zon L.I., Sun W., Johnson G.L. (2001). Role of MEKK2-MEK5 in the regulation of TNF-alpha gene expression and MEKK2-MKK7 in the activation of c-Jun N-terminal kinase in mast cells. Proc. Natl. Acad. Sci. USA.

[86] Timokhina I., Kissel H., Stella G., Besmer P. (1998). Kit signaling through PI 3-kinase and Src kinase pathways: An essential role for Rac1 and JNK activation in mast cell proliferation. EMBO J..

[87] Wang B., Tsukada J., Higashi T., Mizobe T., Matsuura A., Mouri F., Sawamukai N., Ra C., Tanaka Y. (2006). Growth suppression of human mast cells expressing constitutively active c-kit receptors by JNK inhibitor SP600125. Genes Cells.

[88] Guma M., Kashiwakura J., Crain B., Kawakami Y., Beutler B., Firestein G.S., Kawakami T., Karin M., Corr M. (2010). JNK1 controls mast cell degranulation and IL-1{beta} production in inflammatory arthritis. Proc. Natl. Acad. Sci. USA.

[89] Niyonsaba F., Ushio H., Hara M., Yokoi H., Tominaga M., Takamori K., Kajiwara N., Saito H., Nagaoka I., Ogawa H. (2010). Antimicrobial peptides human beta-defensins and cathelicidin LL-37 induce the secretion of a pruritogenic cytokine IL-31 by human mast cells. J. Immunol..

[90] Azzolina A., Guarneri P., Lampiasi N. (2002). Involvement of p38 and JNK MAPKs pathways in Substance P-induced production of TNF-alpha by peritoneal mast cells. Cytokine.

[91] Yu Y., Huang Z., Mao Z., Zhang Y., Jin M., Chen W., Zhang W., Yu B., Zhang W., Alaster Lau H.Y. (2016). Go is required for the release of IL-8 and TNF-alpha, but not degranulation in human mast cells. Eur. J. Pharm..

[92] Navines-Ferrer A., Serrano-Candelas E., Lafuente A., Munoz-Cano R., Martin M., Gastaminza G. (2018). MRGPRX2-mediated mast cell response to drugs used in perioperative procedures and anaesthesia. Sci. Rep..

[93] Shtessel M., Limjunyawong N., Oliver E.T., Chichester K., Gao L., Dong X., Saini S.S. (2020). MRGPRX2 Activation Causes Increased Skin Reactivity in Patients with Chronic Spontaneous Urticaria. J. Investig. Derm..

[94] Kay A.B., Clark P., Maurer M., Ying S. (2015). Elevations in T-helper-2-initiating cytokines (interleukin-33, interleukin-25 and thymic stromal lymphopoietin) in lesional skin from chronic spontaneous (‘idiopathic’) urticaria. Br. J. Derm..

[95] Siiskonen H., Harvima I. (2019). Mast Cells and Sensory Nerves Contribute to Neurogenic Inflammation and Pruritus in Chronic Skin Inflammation. Front. Cell Neurosci..

[96] Ando T., Xiao W., Gao P., Namiranian S., Matsumoto K., Tomimori Y., Hong H., Yamashita H., Kimura M., Kashiwakura J. (2014). Critical role for mast cell Stat5 activity in skin inflammation. Cell Rep..

[97] Arifuzzaman M., Mobley Y.R., Choi H.W., Bist P., Salinas C.A., Brown Z.D., Chen S.L., Staats H.F., Abraham S.N. (2019). MRGPR-mediated activation of local mast cells clears cutaneous bacterial infection and protects against reinfection. Sci. Adv..

[98] Pundir P., Liu R., Vasavda C., Serhan N., Limjunyawong N., Yee R., Zhan Y., Dong X., Wu X., Zhang Y. (2019). A Connective Tissue Mast-Cell-Specific Receptor Detects Bacterial Quorum-Sensing Molecules and Mediates Antibacterial Immunity. Cell Host Microbe.

[99] Green D.P., Limjunyawong N., Gour N., Pundir P., Dong X. (2019). A Mast-Cell-Specific Receptor Mediates Neurogenic Inflammation and Pain. Neuron.

[100] Gilfillan A.M., Rivera J. (2009). The tyrosine kinase network regulating mast cell activation. Immunol. Rev..

