# Supplementary material for: Thymic Stromal Lymphopoietin Promotes MRGPRX2-Triggered Degranulation of Skin Mast Cells in a STAT5-Dependent Manner with Further Support from JNK
Source: Cells. 2021 Jan 8;10(1):102. doi: 10.3390/cells10010102 (PMC7826995; doi:10.3390/cells10010102)
Supplement: Supplementary file 1 [file cells-10-00102-s001.pdf]

Thymic Stromal Lymphopoietin Promotes MRGPRX2-Triggered Degranulation of Skin Mast Cells in a STAT5-Dependent Manner with Further Support from JNK

Magda Babina *, Zhao Wang, Kristin Franke and Torsten Zuberbier

Mast cell biology unit, Department of Dermatology and Allergy, Charité–Universitätsmedizin Berlin, corporate member of Freie Universität Berlin, Humboldt-Universität zu Berlin, and Berlin Institute of Health, 10117, Berlin, Germany
* Correspondence: magda.babina@charite.de; Tel.: +49-30-1751649539; Fax: +49-30-450518900

Supplementary Materials:

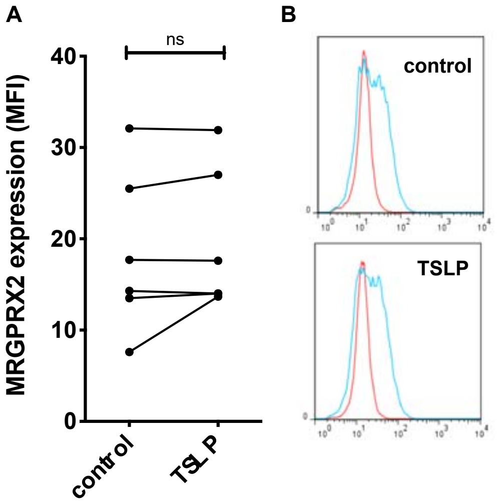

Figure S1. TSLP priming does not change MRGPRX2 surface expression. MRGPRX2 expression at the cell surface was assessed after 30 min with TSLP (7.5 ng/mL) versus medium (control) by flow-cytometry. (**A**) Cumulative results from n = 6 independent experiments (cultures), (**B**) Representative histograms of A, Red: Isotype control. Blue: MRGPRX2-specific antibody. ns, not significant.

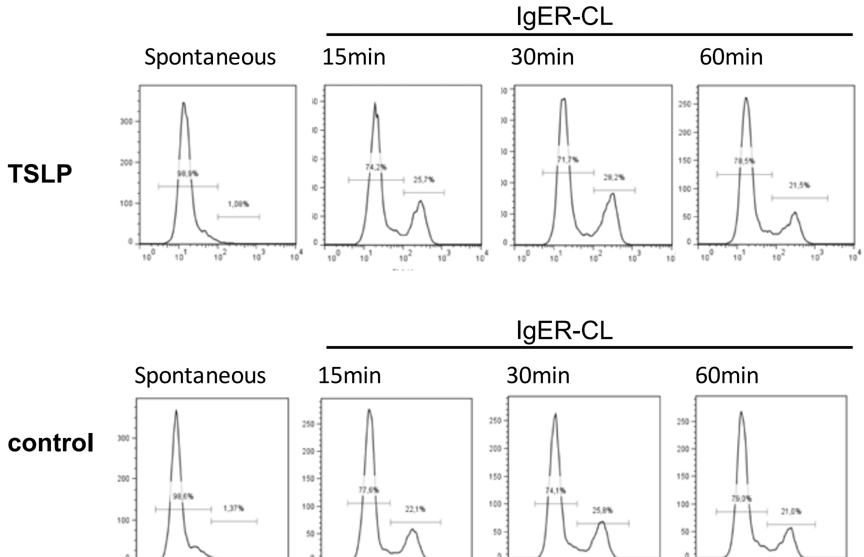

Figure S2. TSLP priming has no impact on the time course of CD107a exteriorization after FcεRI aggregation. MCs were pre-treated with or without TSLP (7.5 ng/mL), then stimulated by IgER-CL (cross-linking) (AER-37, 0.2 μg/mL) for the times indicated. Surface CD107a expression was detected by flow-cytometry. Note that

CD107a exteriorization is delayed after FcεRI aggregation compared to MRGPRX2 ligation (detectable after 15–60 min), in accordance with the two patterns of granule discharge [24]. One out of two independent experiments is shown.

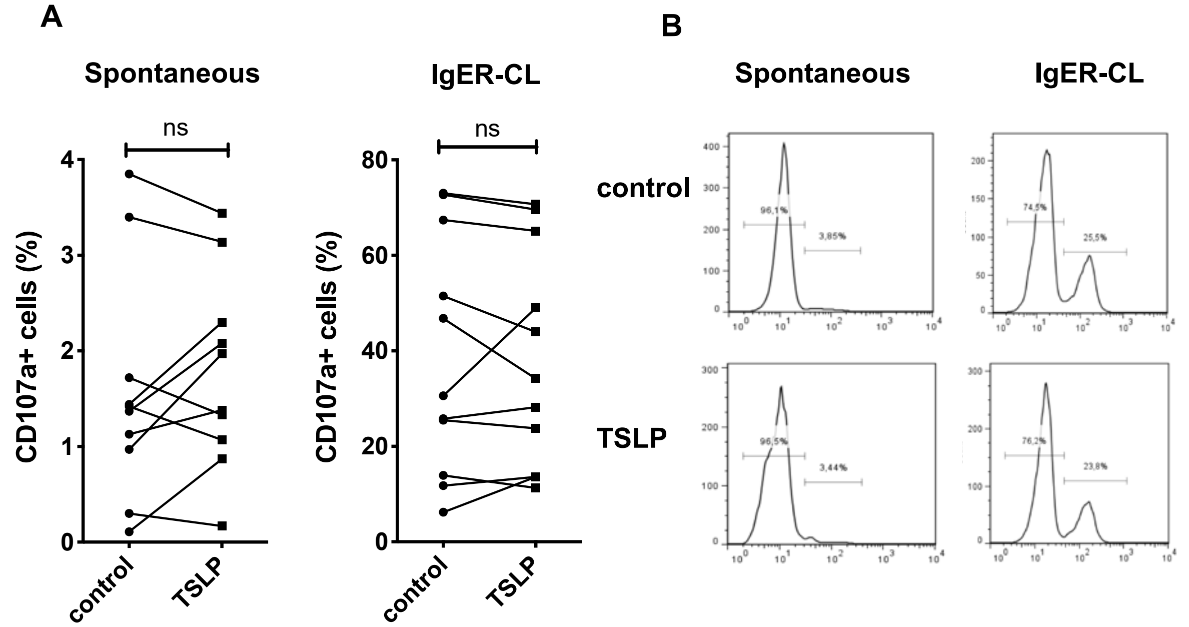

Figure S3. No effect of TSLP on the spontaneous or FcεRI-elicited proportion of CD107+ cells in multiple MC preparations. MCs were pre-treated with or without TSLP (7.5 ng/mL), then stimulated by IgER-CL (cross-linking) (AER-37, 0.2 µg/mL) for 30 min or kept in buffer alone (spontaneous). (**A**) CD107a cell surface expression by flow-cytometry as interconnected dots of 10–11 independent experiments. (**B**) Representative histograms of A. ns, not significant.

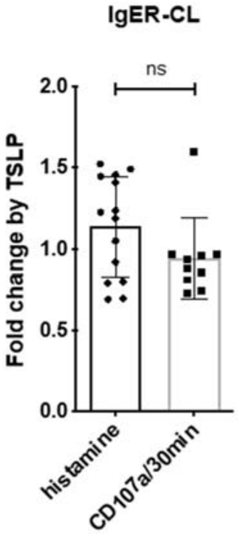

Figure S4. Comparison between FcεRI-triggered histamine release and CD107a exteriorization in the presence of TSLP. The effect of TSLP (7.5 ng/mL) was compared between histamine release (according to Figure 1) and CD107a exteriorization (according to Figure S3), as in Figure 3. The data are presented as fold change by TSLP against control calculated separately for each individual experiment. Mean ± SD of n = 10–14 independent experiments. Ns, not significant.

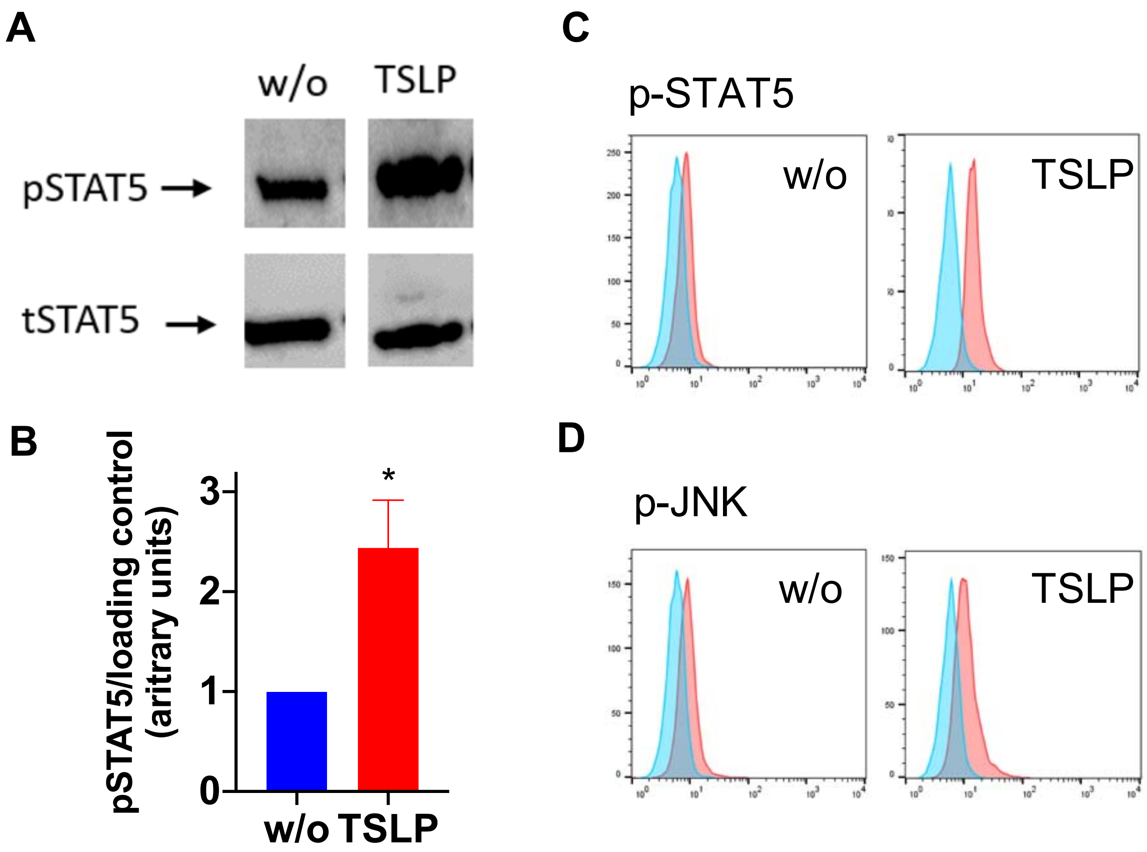

Figure S5. TSLP elicits phosphorylation of STAT5 in skin MCs. A, B) MCs were treated for 30 min with TSLP (7.5 ng/mL) or without (w/o) TSLP. Blots were performed as described in the Methods section and band intensities quantified. (**A**) Representative experiment, (**B**) Normalized pSTAT5 signal as mean ± SEM of 5 independent experiments. **C**, pSTAT5 and (**D**) pJNK were measured by flow-cytometry without and after TSLP (7.5 ng/mL, 30 min) treatment, as detailed in Methods. Blue: Isotype control. Red: antibody against phosphorylated signaling component (given above the histogram). Representative of n = 5. * $p < 0.05$.

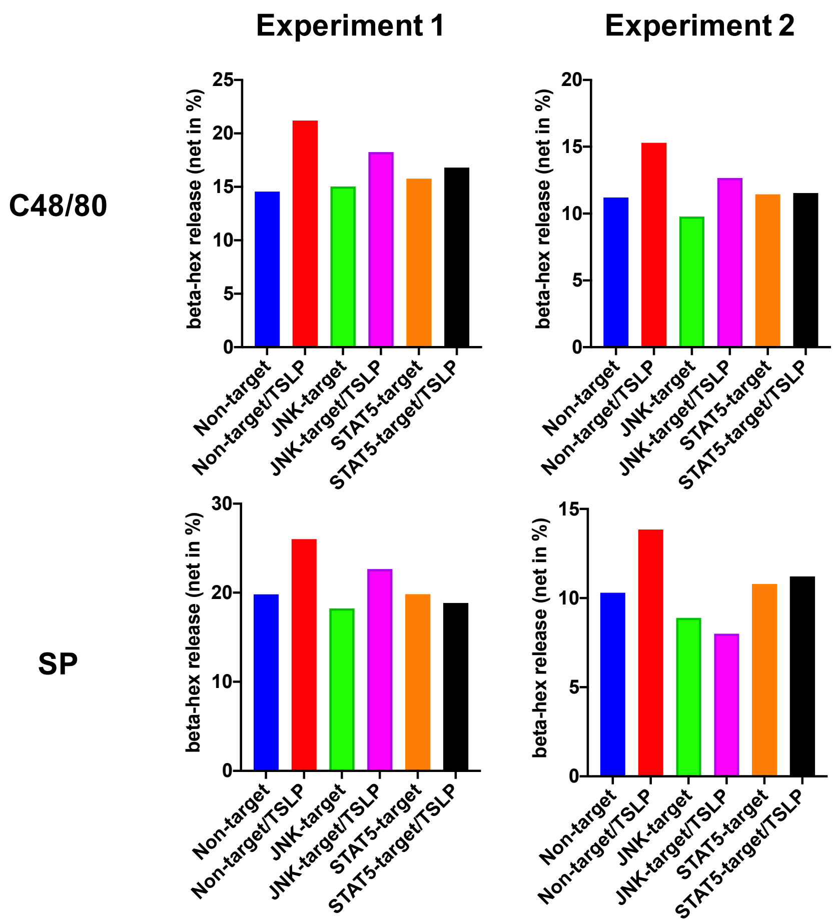

Figure S6. STAT5 is essential for TSLP to support MRGPRX2-mediated degranulation—insights from RNAi-mediated knockdown. MCs were subjected to RNA interference as described in Methods for 2 d, after which time cells were pre-treated or not with TSLP (7.5 ng/mL) prior to being stimulated by c48/80 (10 μg/mL) or SP (30 μM). Beta-hex(osaminidase) release was assessed after 60 min, spontaneous release was quantified analogously, and net release calculated. Net release values from two separate experiments (MC cultures) are depicted separately for each pretreatment/stimulus combination. The data served to calculate the "with TSLP/without TSLP" ratios depicted in Figure 6.
